# Parental Perspectives on Communication from Health Care Providers following a Newborn Diagnosis of Congenital Cytomegalovirus Infection: A Secondary Analysis of a Qualitative Study

**DOI:** 10.3390/ijns9030049

**Published:** 2023-08-27

**Authors:** Tatiana M. Lanzieri, Mary Ann K. Hall, Ashrita Rau, Holly McBride, Danie Watson, Carol Rheaume, Gail Demmler-Harrison

**Affiliations:** 1 Division of Viral Diseases, National Center for Immunization and Respiratory Diseases, Centers for Disease Control and Prevention, 1600 Clifton Road, Mailstop A-34, Atlanta, GA 30333, USA; 2 Division of Hospital Medicine, Emory University School of Medicine, 101 Woodruff Cir, Atlanta, GA 30322, USA; 3 Department of Pediatrics, Baylor College of Medicine, 6701 Fannin St., Houston, TX 77030, USA; 4 Kirby Marketing Solutions, 3808 Villas Del Sol Ct, Tampa, FL 33609, USA

**Keywords:** congenital cytomegalovirus, newborn screening, communications, qualitative study

## Abstract

The study objective was to identify communication messages that parents of children diagnosed with congenital cytomegalovirus (cCMV) infection reported as essential and helpful. We performed a secondary analysis of focus groups and interviews conducted with 41 parents of children with cCMV who had enrolled in a long-term follow-up cCMV study at an academic medical center. Three groups of parents who had children with cCMV participated in the study: parents with children symptomatic at birth, parents with children asymptomatic at birth who later developed sensorineural hearing loss, and parents with children asymptomatic at birth who remained asymptomatic into adulthood. Using a health marketing approach, we identified six general themes from the focus group sessions: initial diagnosis, likely health outcome(s), comfort and coping, symptom watch, resources, and prevention. Receiving the initial diagnosis was shocking for many parents, and they wanted to know how their child would or could be affected. They valued access to the information, follow-up visits for early detection of hearing loss and other developmental delays, and support from other parents. Parents wished to obtain this information from their pediatrician but felt that experts offered more up-to-date knowledge about prognosis, monitoring, and treatment. With more U.S. states implementing cCMV screening strategies which would lead to more infant diagnoses, it will be necessary for providers to meet parents’ expectations and communication needs.

## 1. Introduction

Congenital cytomegalovirus (cCMV) infection occurs in 4.5 per 1000 live birthed infants in the United States [1]. Approximately 10% of infected infants exhibit clinical findings at birth and 10–15% of the asymptomatic infants present with sensorineural hearing loss during early childhood [2]. The remaining 75–80% of infants with cCMV do not appear to develop disease or sequelae [3]. However, a recent study has shown that vestibular, gaze, and balance disorders may occur in 45% of asymptomatic infants but their clinical impact has not been assessed [4].

Despite the serious consequences that can result with cCMV, studies have reported low knowledge and frequency of cCMV counseling among obstetric specialists and, among pediatric general providers, mixed knowledge and low comfort with caring for children with cCMV [5,6]. Other studies have reported that knowledge about cCMV is low amongst expectant parents [7,8], and that parents of children who have cCMV emphasized that they would have benefitted from clear guidance and support post-diagnosis and information about cCMV prevention during pregnancy [9].

With a growing number of U.S. states implementing cCMV screening, more infants will be diagnosed with cCMV and will need continued care. Parents of these infants will need guidance and clear messaging from their healthcare providers. It will be important for providers to meet parents’ expectations and communication needs. In this study, we performed a secondary analysis of qualitative data from focus groups and interviews conducted in 2013 with parents of grown children who were identified with cCMV infection in the newborn period and enrolled in a longitudinal study at an academic medical center.

## 2. Materials and Methods

A qualitative study using multiple focus groups and interviews was conducted in September 2013; participants were a convenience sample of parents of children with cCMV enrolled in a longitudinal study at Baylor College of Medicine, who agreed to participate in the study, as previously described [10]. The primary objective of the study was to assess perceptions of the utility and acceptability of newborn CMV screening among parents of children who screened CMV positive or were diagnosed with cCMV and were followed through adolescence, as previously described [10]. We followed the Standards for Reporting Qualitative Research (SRQR) recommendations [11]. The Cytomegalovirus (CMV) Study offered patients and families regular consultation with cCMV experts and follow-up assessments from infancy to adolescence: at ages 4–6 weeks, 4–6 months, 9–12 months, 18–24 months, approximately yearly through elementary school, and then every 2–3 years through high school [10]. Monitoring and follow-up evaluations for children who were asymptomatic at birth were more frequent and rigorous than the typical follow-up would have been outside of a research setting.

Parents of children who were born with cCMV were recruited from three groups: (1) parents whose child was born symptomatic and were identified through referrals rather than screening, and who typically presented with sensorineural hearing loss or intellectual disability (*n* = 12); (2) parents whose child was born asymptomatic but who subsequently developed sequelae (typically sensorineural hearing loss) (*n* = 11); and (3) parents whose child was born asymptomatic and who never developed disease sequelae (*n* = 19).

Focus groups and interview guides (Appendix A and Appendix B; https://omb.report/omb/0920-0970 (accessed on 5 July 2023) were developed based on a review of scholarly articles and the CMV Study materials to assess the psychosocial impact of newborn screening for cCMV, the primary goal of the study [10]. Focus groups and interviews were conducted separately for the three groups and facilitated by two trained and experienced moderators known to the participants. They included similar questions across all three parent groups with some modifications to reflect experiences with disease sequelae. Due to the size of the focus groups, parents whose children remained asymptomatic were afforded a slightly different opportunity to provide message content information by writing down their top three messages for new parents. There was a wide range of participating family members: mothers were the most frequent participants (76%), but fathers, stepparents, and one grandmother also participated. The demographic characteristics of the children whose parents participated in the qualitative study are described in Table 1; most were white (76%), had married parents (62%), and approximately half (47%) had high socioeconomic status. The focus groups and interviews took place in a time frame where parents had ample opportunity to reflect upon their communication needs: children of participants were a mean 23 years old (SD = 5.2 years) and had spent 22 years (SD = 3.7) in the CMV Study on average [10]. Parents gave oral consent to participate, and the institutional review board of Baylor College of Medicine approved the research.

Focus groups were conducted in person and lasted about 120 min. Interviews were conducted in person or by phone and lasted approximately 90 min. All focus groups and interviews were recorded, transcribed, verified for accuracy, and imported into QSR NVivo^©^ qualitative data analysis software Version 10 without personal identifiers. Transcripts were thematically coded by two coders. One coder was also the moderator of the focus groups and interviews. The other coder was a researcher with experience in qualitative analysis who had not participated in the interviews or focus groups. We used a combination of deductive and inductive strategies to code transcripts and identify emergent themes. We assessed the newborn screening literature in order to situate the data within the larger context of the literature, provide possible themes we expected to find in the focus groups and interviews, and to check for possible coder biases by using other sources to verify categories and themes [12]. We began by using a deductive approach, informed by the data generated from the literature review. We examined transcripts for the possible themes and then re-examined transcripts for any new or different themes that we had not seen in the literature. The process of using both deductive and inductive reasoning allowed us to use existing literature and also look for new themes not yet discovered. Coders met regularly to discuss coding categories, examine and rectify discrepancies, and adjust definitions of thematic codes (results are presented below, organized by the overarching themes identified in analysis). For coding passages related to communications around cCMV diagnosis, we used a health marketing approach. Themes that occur with enough frequency in the health marketing field are typically ones that are recommended for use in practice. We identified message content and sources that parents reported as important to them. We looked at the data across the three parent groups and then searched for differences in a parent group that could be strong enough to warrant a tailored and group specific approach in communication. This is a common approach in health marketing referred to as segmenting the audience and tailoring the messages. However, due to little difference in responses between the three groups in the initial analyses, we aggregated the data for messaging across all parent groups.

## 3. Results

### 3.1. Content

There was a wide range of messages, with some commonalities across parents. The most frequently shared messages that participants felt would be helpful for families of children newly diagnosed with cCMV are shown in Table 2. The message content obtained from the focus groups included six general themes: initial diagnosis, likely health outcome(s), comfort and coping, symptom watch, resources, and prevention.

### 3.2. The Initial Diagnosis

Parents reported feelings of shock and dismay at receiving a cCMV diagnosis for their child; all expected to bring home a healthy child, and unexpectedly learning of an infection that could result in a potentially serious medical problem was highly distressing. Parents felt that a balanced message at the initial diagnosis that explained what CMV is, and what it is not, would be helpful for parents in similar situations. For example, parents who felt the news was delivered to them relatively well said their doctor told them their child tested “positive for a virus, a rather common virus, something like the common cold”. Simply initiating the diagnosis conversation this way appeared to have made a big difference in helping parents cognitively process the diagnosis. Parents wanted to be provided with basic information at the initial diagnosis, including but not limited to the definition of CMV; how CMV is transmitted, and how a fetus or infant contracts it; the health problems cCMV causes most commonly, how it causes them, and how long the problems last; and the risks to other children inside and outside the household and related prevention measures, especially prevention for other pregnant women and newborns. Even though parents said they were initially overwhelmed or shocked by the news, they wanted the truth, accompanied by comforting messages of hope, such as “every child is unique, and these problems are unlikely to develop in your child”.

Parents stated that the most difficult time for providers to speak with them about their child’s CMV status was immediately after childbirth. Parents often commented that the news about cCMV was difficult to process at that time they were exhausted from labor. Instead, parents suggested that providers assess how urgent it is to pass on the cCMV news and conduct any other tests, and use that information to inform the timing of the diagnosis discussion.

Once they received the diagnosis of cCMV, parents often had many questions arise in quick succession. If the notification of the diagnosis takes place in person, many parents suggested providing information in writing (and in pictures), and/or offering writing paper for parents to take notes. Parents greatly preferred in-person notification versus a phone call. If the notification must be by phone, pointing parents to a website with information can help to guide them in understanding their child’s cCMV diagnosis and their own research.

### 3.3. The Likely Health Outcomes

Parents wanted to know how their child would be affected by cCMV in both the short and long term. Many said they wanted to know the best- and worst-case scenarios immediately upon receipt of the diagnosis—however, they noted that because information at this stage could be lost due to poor processing ability, exhaustion, and fear, providing an overview of the most likely outcomes with access to information about other less likely outcomes might be a better strategy.

Parents liked the idea of accessing the information in a staged (by age or by milestones) approach. Parents also stated that, by and large, they wanted an “honest assessment or prognosis for their child”. However, some pointed out they wanted a balanced positive and negative outlook. Some felt doctors only gave them the negative prognosis and they only perceived any potential for positive outcomes for their child when they entered the CMV Study.

Parents felt that the best advice they received was to “stay positive for your child”. Positivity was particularly encouraged among parents of asymptomatic children, who stressed the importance of being reminded that as their children grow, their chance of experiencing a negative health outcome is low, while also highlighting the necessity of encouraging follow-up testing to help identify any adverse outcome early. Many years after having their child go through follow-up, parents across all groups expressed that they now understood the value and utility that the follow-up has for child development and parenting skill.

### 3.4. Comfort and Coping

Whether the child was asymptomatic or symptomatic at birth, coping and comforting messages were perceived as instrumental in helping parents function well after the initial diagnosis and continued to be essential for long periods of time. Caring medical staff and clear information were regarded as important mediators to the potential negative stressors and psychosocial impacts of dealing with a cCMV diagnosis.

Across all parents, the most common message they thought would help new parents cope was to hear messages about the power of knowledge and of educating oneself about cCMV, and then monitoring your child. Parents also wanted new parents to receive messages about staying positive and hopeful for their child and to enjoy and love their child. All parents were vocal about proactively completing follow-up testing, particularly parents of children who were asymptomatic at birth and subsequently developed sequelae. A parent admitted that they thought everything was fine and then, “one time we came in for a test and she had lost 40% of hearing in one ear. I was so thankful we caught it early”.

Parents whose children were symptomatic at birth appeared to experience more shock, anxiety, grief, and fear upon initial diagnosis than parents in the other two groups. Some messages suggested by parents of asymptomatic children would be inappropriate for parents of symptomatic children. These messages included reassuring parents that it was likely that their children would not experience any sequalae, but to work closely with their pediatrician and do the follow-up testing to catch anything early. Parents of children who developed sequelae and needed child development or hearing interventions suggested messages tailored specifically for how to communicate effectively with schools and teachers to help them accommodate their child’s needs.

Finally, in all groups, parents emphasized the importance of remaining optimistic, noting that there were and are many excellent treatment options available for hearing loss, and there are varying levels of hearing loss that children with cCMV may experience; being open about cCMV, and teaching your child how to live with it; and making a plan, a step-by-step process of what will help your child most.

### 3.5. Symptom Watch

Parents felt it was important to be educated about all symptoms to watch for, how to monitor for symptoms at home, and how to help teachers or childcare workers monitor for the symptoms as well. Several parents mentioned they were concerned that continuing to monitor children year after year, despite not finding symptoms in the early years, would be daunting for new parents. Many parents suggested that having their clinician provide a checklist of symptoms to watch for, by age, such as a “road map” to aid parents in knowing what to monitor and test for in the coming year, would have been very helpful to them. They suggested that such a document could contain a plan that would describe symptoms in a checklist fashion but would also explain which tests would be coming up, at which ages, what the tests measure, and why they are important. This document would consequently allow for clinicians to give parents concrete advice and a timeline of action steps. Parents also repeatedly reiterated that they perceived communicating the need for symptom watching to be extremely important to prevent unnecessary delays in language and intellectual development.

### 3.6. Resources

Parents stated that they considered information from trusted and reliable sources as crucial. In terms of clinical support, parents stated that a comprehensive program to conduct initial and follow-up testing for the children (such as the CMV Study) and provide facilitated parent support and feedback (e.g., in formal support groups) is needed, even for asymptomatic children.

In terms of broader social assistance, several parents of cCMV children described the vital social support they received from their own parents, and they wondered aloud how young, isolated parents could manage this situation without that support. They felt that young parents in particular could benefit from formal or informal support groups (even if virtual), especially those who do not live near their own parents.

Parents also recommended reaching out to other parents of children with cCMV, especially those with similarly diagnosed children. Parents whose children are at similar stages or experiencing similar tests or treatments had served as good support systems for these parents. The anxiety and fear were still present each year during regular follow-up periods. “Other parents, like you, can help lend a friendly ear and help you through your fear. They know what it’s like,” as one parent put it.

Parents of children with sequelae also appreciated referrals to specialists, such as neurologists and audiologists, who were experienced with and knowledgeable about CMV.

### 3.7. Source and Channels

Parents wished to obtain support and knowledge from their existing pediatrician, with whom they had already developed a relationship. However, parent participants felt that most pediatricians were not knowledgeable about cCMV, possible prognoses, testing, or treatment protocols. Parents felt that CMV experts offered more up-to-date scientific knowledge about treatment options, testing procedures, new innovative options, and the prognosis for children diagnosed with cCMV.

Parents wanted their healthcare providers to help them navigate the online world by suggesting the most trusted websites for accurate and reliable information. They felt it was important to be provided with both accurate information and a caring medical approach that offered opportunities to have follow-up dialogue with providers willing to answer questions that might arise from Internet-based information-seeking. Parents stressed that this dialogue with healthcare providers was particularly important during the child’s first few years of life.

Parents also felt that brochures, checklists, child-specific plans, and newsletters from their health care providers were important channels for delivering information and education to new parents. Mothers in particular wanted some simple, frequently asked questions (FAQ) documents they could share with their spouses, family, and friends. Parents reported occasional difficulty navigating conversations about their cCMV child with friends and family, which could become highly emotionally charged.

### 3.8. Prevention

All parents voiced strong support for efforts to increase public awareness of cCMV so that family, friends, and faith communities could provide needed support to families. When parents received good social support, it had powerful and positive effects on their own mental and emotional health.

## 4. Discussion

This qualitative study among parents of children identified with cCMV infection brings their perspective on communication messages that were important and most helpful to them following a cCMV diagnosis and thereafter. Parents wanted to know how their child would be (or was likely to be) affected, and valued access to the information in a staged approach (e.g., by age, or by milestones); to follow-up for early detection of hearing loss and other developmental delays; and to support from other parents.

### 4.1. Communication of Clinical Information from Providers

Although parents wished to obtain support and knowledge from their pediatrician, they felt that CMV experts offered more up-to-date knowledge about treatment, monitoring, and prognosis. As more U.S. states are moving toward newborn screening for CMV, lack of knowledge among general pediatricians is a significant gap, and general pediatricians may need to become more conversant with all aspects of newborn screening, including follow-up confirmatory testing, monitoring, and treatment guidance.

It is important for healthcare providers to clearly communicate with parents, particularly because parents are likely to experience significant uncertainty about potential long-term outcomes for children diagnosed with cCMV. Parents of children with asymptomatic cCMV need to be reminded by their healthcare providers that the odds their child will develop sequelae are low to reduce distress and anxiety, and that numerous interventions exist for CMV-related hearing loss and developmental delays [13,14,15]. Clear guidance would be needed about follow-up tests and anticipatory monitoring, as well as an informed discussion on the potential benefits and risks of antiviral treatment [16]. This guidance could be supplemented by recommendations for trusted websites, along with other materials (e.g., brochures, checklists, child-specific plans) and newsletters which could provide important channels for delivering information to new parents. In addition, messages should conform to current standards in health communications and be developed with cultural awareness, following best practices in literacy, health literacy, and communications for populations with Limited English Proficiency.

Our findings are consistent with previous research highlighting the importance of clear guidance and support for parents who are raising a child with cCMV because of the potential for significant effects on child development [10,16]. Although most children diagnosed with cCMV through newborn screening will have typical development [17], children with cCMV who present with symptomatic disease at birth may have poorer school performance and delays in expressive language and general development compared to children with similar clinical signs who are negative for CMV at birth [18,19].

Our findings are also consistent with those found in a similar study in the United Kingdom [9], where parents of children with cCMV expressed the importance of knowledge and information, and supported raising awareness around CMV. Notably, parents in our study also voiced strong support for efforts to increase public awareness of CMV, especially on cCMV prevention. As in studies addressing other newborn screening conditions [13], parents preferred receiving information prenatally. A recent study in the United Kingdom found that film-based resources successfully increased knowledge of CMV among new expectant parents [20]. An ongoing study in the United States will assess whether behavioral intervention for susceptible pregnant women can lead to a behavioral change that could decrease primary CMV infection, as well as whether it is acceptable by clinic staff and feasible to implement as part of routine clinical prenatal care [21]. These studies will be important to inform preventative efforts, particularly as more U.S. states enact legislation on CMV awareness and prevention [22].

### 4.2. Social and Emotional Supports for Parents

Parents of children with cCMV who have long-term impairment also may have a lower quality of life and more physical and cognitive issues as compared to parents of children without cCMV infection [18]. Access to information in a staged approach and mental health counseling could have significant impacts on parental and child quality of life, and could aid parents in understanding their child’s needs and areas in which their child may need extra support.

Parents whose children were symptomatic at birth appeared to experience more shock, anxiety, grief, and fear upon initial diagnosis than parents of children who were asymptomatic at birth (with or without later-onset sequelae), and such individuals may benefit from mental health counseling immediately upon receiving the news and in the weeks following.

### 4.3. Limitations

This study has important limitations. This study has a limited number of quotes because it reports on secondary findings on parent’s expectations and communications needs that were evidenced during a qualitative study to assess perceptions of the utility and acceptability of newborn CMV screening. Participants tended to be highly educated and were recruited from a convenience sample of parents of children enrolled in the CMV Study. It is unclear how results might have differed among parents with lower education levels. The majority of participants were also white (76%) and consequently not representative of the general population. We were not able to compare the parents that participated in the qualitative study to those who did not. However, we recruited parents from three groups according to their children’s status (symptomatic, asymptomatic with sequelae, and asymptomatic without sequelae), to reflect their long-term perspective and lived experience. The long-term outcomes of the children participating in the CMV Study were described elsewhere [17,23,24], and likely impact the way parents reported their experience. If parents had been interviewed after receiving initial diagnosis or during follow-up, their perceptions could have been different. Recall bias was possible, as parents described events that occurred years and decades previously. Finally, the study was conducted more than a decade ago with parents whose children were born in the 1980s to early 2000s. These parents may have had different experiences than parents of children diagnosed with cCMV in more recent years. Treatment and interventions for children with cCMV have changed, adding on time sensitivity to discuss diagnostic testing and use of antivirals. Additionally, though general public and provider awareness continues to remain low [5,7,8,25], several U.S. states have enacted legislation to increase CMV awareness, implement hearing-targeted CMV screening, or both, starting in 2013 [23]. Further research is needed to understand parental perceptions and communication needs within the context of more contemporaneous screening programs for cCMV.

## 5. Conclusions

Our findings on the parental perspectives on cCMV communication messages might be useful for health departments in states that have enacted laws or are implementing activities on cCMV education, prevention and screening [22], to increase awareness and education among health care providers, expectant or new parents, and the public. Even though the parents included in this study had received their child’s cCMV diagnosis years or decades prior to their participation in the study, their perceptions that their general health care providers were not up to date on cCMV knowledge, treatments, and prognosis are consistent with findings of more recent surveys among healthcare providers [6,25]. As more U.S. states move to implementing newborn screening for cCMV, general pediatricians may need to become more facile with all aspects of newborn screening, including follow-up confirmatory testing, monitoring, and treatment guidance.

## Figures and Tables

**Table 1:** Demographic characteristics of children diagnosed with cCMV whose parents participated in focus groups and interviews.

Characteristic	Number (%)
Number of interviewees per child	
One parent/guardian	26
Two parents/guardians	8
Total children, total interviewees	34, 42
Parent/guardian interviewed ^a^	
Mother	32 (76)
Father	3 (7)
Step- or foster parent	3 (7)
Grandmother	1 (2)
Unknown	3 (7)
Marital status of parents at time of interview ^b^	
Married	21 (62)
Divorced or separated	9 (26)
Widowed	1 (3)
Unknown	3 (9)
Child’s gender ^b^	
Female	14 (41)
Male	20 (59)
Child’s race and ethnicity ^b^	
Non-Hispanic, White	26 (76)
Hispanic, White	3 (9)
Non-Hispanic, Black	5 (15)
Parents’ socioeconomic status at time of child’s birth ^b^	
High	16 (47)
Medium	13 (38)
Low	5 (15)

^a^ Percentages based on the number of parents/guardians who were interviewed; ^b^ Percentages based on the number of children whose parents/guardians were interviewed.

**Table 2:** Helpful messages for health care providers to communicate with parents of infants newly diagnosed with cCMV identified in focus groups and interviews with parents of children with cCMV.

Content	Messages	Supporting Parental Quote
***Communications from health care providers***		
1. Initial CMV Diagnosis	Explain the CMV basics, what is CMV, how you get it, who gets it, can you give it others. Explain that it’s common in the population, like a cold. Say it’s not exotic. Explain how it can be prevented for other members of their family and friends.	Parents who felt they were delivered the news well said their doctor told them their child tested “*positive for a virus, a rather common virus, something like the common cold*.”
	Explain the likely health problems, including the best- and worst-case scenarios and a brief description of the prognosis as best known at that point in time (see # 2).	Parents stressed the importance of knowing likely health problems, and that “*the benefits could be that you get to know if there is progress, regression, what areas should you work on to improve the quality of his life and areas that you wouldn’t have to focus on as much by having this information, and also the health or knowledge of how is he doing, what are we expecting.*”
	Provide coping and comforting messages (see # 3).	Comforting messages of hope included “*every child is unique, and these problems are unlikely to develop in your child.*” Parents of asymptomatic children explained that these comforting messages also included best-case scenarios such as “*[there is] a 90% chance of nothing happening.*”
	Tell parents they will be getting a list of symptoms to watch for and monitor in the future (see # 4).	This awareness was “*priceless*” for parents who felt that it gave them “*just where to start when you feel so lost. It was like the beginning of a map.*”
	Provide all information in writing so parents can refer to it later and share with others. Post on the Internet also.	Parents described wanting to consult a “*website [they] could trust*” and wanted information in writing as they realized they “*wouldn’t remember anything*” from the initial conversation.
	If child is symptomatic, offer mental health counseling and resources to parents experiencing significant stress, grief and fear during this stressful time.	Parents emphasized that they needed help coping with the stress and grief of realizing that their child was not a “*perfect little baby.*” They described the difficulty of being confronted with the knowledge that “*this could be a lifelong challenge.*”
2. Likely Health Outcome(s)	Explain the range of health outcomes parents can expect, from best- to worst-case scenario. All of it now. (Some mothers cautioned against overwhelming those women who are exhausted from childbirth.)	Parents wanted an “*honest assessment or prognosis for their child.*” Parents of asymptomatic children also stressed that they wanted medical providers to “*reassure them that it is highly likely nothing will happen.*”
	Explain the percentages for those who are asymptomatic and never develop health problems, and the percentages for those who do develop health problems.	Parents wanted to hear “*here are the percentages, here’s the worst case, best case.*” They appreciated when medical providers told them “*statistics*” and ”*the likelihood that [their child] was going to have symptoms and what they might be.*”
	Answer the most difficult questions about prognosis, such as will they be developmentally challenged, will they be deaf.	These difficult conversations benefitted parents, who mentioned that “*It prepared me for what could happen. If I hadn’t known then, it would’ve been even more sad.*” Parents also emphasized that difficult conversations were vital because “*not knowing the truth doesn’t make it not true.*”
	Remind them of the benefits of follow-up testing, early identification of testing, child development and parenting skills assistance.	Parents talked about the benefits of follow-up testing and assistance in helping them “*[make sure their child is] on the right track and on schedule.*” They also discussed how follow-up testing is beneficial because of the importance of “*catch[ing] anything early.*”
***Message to other parents***		
3. Comforting and Coping	Knowledge is Power. Educate yourself about what to symptoms watch for and monitor your child.	One father stated: “*We started out the conversation saying that knowledge is enlightenment and that the more that you know the better you are prepared to deal with what is coming and what can be.*” Another parent discussed how being “*attentive*” would allow for them to seek out “*intervention [when their child] is struggling.*”
	Stay positive and hopeful for your child.	One mother reminded parents to “*look at this as an opportunity to fall even more deeply in love with your child.*” Another emphasized that they see their child as “*a person, not as a person with CMV.*”
	Be proactive and complete all follow-up testing.	Parents focused on follow-up testing as allowing them to make sure their child was “*on the right track and on schedule.*”
	Prepare me for what lies ahead One day at a time; one decision at a time.	Parents emphasized the importance of “*a plan, a step-by-step process of what will help your child most.*” They stressed that they needed a “*kind of plan for [their child] that [they] could keep track of.*”
4. Symptom Watch	Keep an eye out for the main symptoms and keep getting them tested, especially for age, stage of development, and critical child development milestones.	Parents explained that just having “*some knowledge about where [their child] was developmental-wise was very helpful.*”
	Remember the important thing is that getting help early is why monitoring symptoms is important. They can do a lot to help your child if they catch it early.	A parent highlighted that monitoring symptoms is vital and discussed how “*one time we came in for a test and she had lost 40% of hearing in one ear. I was so thankful we caught it early.*”
	Separate a typical child problem from real CMV symptom problems. So get educated on CMV symptoms, especially hearing issues, which is the most typical CMV symptom.	Parents suggested that learning about potential symptoms was helpful and that “*the statistics on what is the likelihood that he was going to have symptoms and what they might be… that was very positive.*”
***Resources to share***		
	Provide parents brief information sheets about where to get reliable information and education about CMV and their child. They specifically wanted lists of trusted websites, books, bloggers, and newsletters (e.g., Cleveland Clinic newsletter).	Parents stressed that “*they have to have somewhere to turn to for information.*”
	Let parents know about community or insurance covered comprehensive programs for child testing, treatment, and parent education.	A parent mentioned that they “*would want some sort of program, somebody to handhold me through the ages and stages.*” Another explained the importance of having a program to help them and stated that “*without a program, it would be unconscionable to saddle a new parent with CMV test news.*”
	Let parents know where to find parent support groups—offline and online.	Parents pointed out the benefit of support groups: “*Other parents, like you, can help lend a friendly ear and help you through your fear. They know what it’s like.*”
	Let parents know where to find names of medical specialists experienced in CMV, such as neurologists and audiologists.	Parents emphasized the importance of medical specialists: “*without that support, without the guidance, without the knowledge that is shared from the doctors and from the clinic, I would be absolutely helpless in helping my child and just having the support there means a lot because if there is a need you know that there is someone there with you.*”They explained that medical specialists provided “*honest answers*” and an opportunities to “*get questions answered and settle my concerns.*”

## Data Availability

The data presented in this study are available on request from the corresponding author Tatiana M. Lanzieri (tmlanzieri@cdc.gov). The data are not publicly available due to privacy restrictions.

## Appendix A. CMV Parent In-Depth Interview Guides

**Group 1—Parents of children born with CMV who were symptomatic at birth and may or may not have developed sequelae later on**
**Group 2—Parents of children born with CMV who were asymptomatic and later on developed sequelae**
**[** *variations indicated within brackets* **]**
**Consent**
Good afternoon, my name is _____________. I want to thank you in advance for taking time from your busy schedule to participate in this interview. This research is being sponsored by the Centers for Disease Control and Prevention. The information you provide today will be extremely useful to other parents and to professionals working on CMV. When one of the CMV staff members called and asked you to participate today you were told that this interview is entirely voluntary, that no LAST names will be used today or in our reporting of the data, and that if there are any questions you do not want to answer or feel are too sensitive you are free to not reply. In fact, you are free to leave the interview session at any time. We will be recording today’s session for our note-taking purposes, so please let me know if you prefer we not record the interview. The last important thing to know is that we are aware of your experience with CMV testing, but we do not have access to any of your child’s medical records. If you could signify by saying your FIRST NAME ONLY and YES you understand how your privacy is being protected we can proceed. I’ll stop for a minute to see if you have any questions.
**Purpose**
As I said my name is _________ and I am working with Dr. Gail Demmler-Harrison and her CMV team on a project to help us better understand the kinds of positive and negative mental, emotional, financial, and family issues that parents experience when their child is [*with health problems and then diagnosed with congenital CMV/ first tested for congenital CMV and in the following critical years of development as follow-up tests may be completed*]. One of the best ways for us to do that is to talk directly to people like you who know a lot about those experiences. I will be asking you to share your thoughts, feelings, and opinions about these experiences. Please remember that there are no right or wrong answers—we just want to know about your own thoughts, whatever they may be. From time to time I may move the discussion so we can cover all of our topics tonight. Let me stop and ask if there are any questions so far.
**Procedures**
- Session should last about 45–60 min - Secure– when we report about these conversations later, not even your FIRST name will not be used in the report. - The restrooms are ____. Feel free to get up at any time during our discussion. - Any other questions before we start? Okay, let’s get started.
**Warm Up and Opening Question (5 min)**
1. First, I wonder if you could tell about some of your happiest memories from the first 5 years of life of your child who was born infected with CMV.
[*2. How did you know that your child had health problems?—Group 1 only*]
[*3. How did their diagnosis with congenital CMV occur? Did it happen right away or did it take time before you received a diagnosis?—Group 1 only*]
**Negative Psychosocial Impacts of CMV Test and Follow-up Testing** (Note: moderator will rotate the positive and negative impacts sections among the interview sessions to minimize any order bias].
4. As you know raising a child can have its ups and downs. But we are particularly interested in your memory of experiences you had when your child was first diagnosed positive for congenital CMV. So take a minute and think back now to that time, when your child was an infant and you received this testing news. Tell me a about your first reactions?
5. After the positive CMV test results were given to you, what would you say were the first few things you wanted to know? Was anyone able to answer those questions for you?
6. In those first few days tell me how you felt about yourself in regards to the positive test? [Probe for feeling alone, feeling responsible, being scared, feeling overwhelmed, no major concerns, not a problem].
7. In the first few days that followed the positive CMV test results, tell me about any kinds of family or marital stress you experienced. [Moderator to probe for spousal issues like blaming, concerns about how my other children and how they would cope, worries about what my family would think/react].
*[8. Can you tell me what you thought about in terms of financial issues related to the positive test results? Did you worry about anything, what kinds of things? [Probe for concerns about enough money to have necessary follow-up tests done, concerns about health insurance coverage, worries about how I can care for this child and my other children].—Group 2 only]*
a. What other kinds of things concerned you at that time—say in the first 2–3 months of life?
9. Now let’s talk a little about the years following the initial test results. You participated in the follow-up clinic for some period of time. After the first few months, what kinds of new concerns or stressful issues surfaced? [Probe for stressors specific to self, marriage/spouse, and parenting skills/efficacy].
10. After the first few months, what kinds of new concerns or stressful issues surfaced related to your family, friends, and other social networks (e.g., church)?
[*a. Did any new financial or work-related concerns surface in the first few years of follow up? [Probe for health insurance costs, job loss, work conflicts].—Group 2 only*]
11. Now thinking about the medical professionals you met with over those first few years, were there any additional stressors from those meetings? What kinds of things frustrated you? What could have been explained better? Thinking about new parents today, how would you explain some of the things that frustrated you?
[*12. Thinking about those extra doctor visits and follow-up tests that your child went through, what kinds of stress or negative impacts did those activities or tests have on you and your family? [Probe for transportation hassles of getting to appointments, concerns about time off of work to get to appointments, costs of travel, difficulty with child care for other children].—Group 2 only*]
13. Now let’s talk about your interactions with your child. Thinking back over those first few years, how would you characterize your relationship with your child? [Probe for stressors related to child behaviors, lack of affection, over-protectiveness].
14. If you have other children, did you feel that your relationship with CMV+ child was different in some ways due to the CMV? How so?
a. Did it seem as though your child was about the same or more difficult to parent than other children you knew then? In what ways?
[*b. I understand that your child was generally healthy at birth. At what age was your child diagnosed with hearing loss associated with congenital CMV? How did the diagnosis occur? What was your reaction? Has your child experienced any other health problems associated with congenital CMV?—Group 2 only*]
[*c. Did knowing that your child had congenital CMV make it easier or harder to deal with the diagnosis of the hearing loss or other health problems? In what ways?—Group 2 only*]
15. As your child got older, what kinds of concerns did they have about getting the doctor visits and follow-up tests done? What could have been done or said to you to improve that experience for you and for your child?
**Positive Psychosocial Impacts**
(Note: moderator will rotate the positive and negative impacts sections among the interview sessions to minimize any order bias. Now let’s switch gears and talk about some of the positive aspects of having the CMV test done and the knowledge that came with that.
[*16. In what ways was it helpful to know that your child’s health problems were caused by congenital CMV?—Group 1 only*]
[*17. Thinking back to initial diagnosis of congenital CMV infection in your newborn, what would you say was the most positive aspect of getting those results?—Group 2 only*]
[*18. Now thinking about the following few years and the follow up tests that you and your child went through, what would you say are the best things about having those follow-up tests done? [Probe for the kinds of concerns that the follow-up testing minimized].—Group 2 only*]
[*19. All in all, tell me about your thinking on the value of CMV testing and follow-up tests. Were the tests worth the troubles, stress, and issues they caused for you as a parent? Do you wish your child had never been tested? Why or why not?—Group 2 only*]
[*a. What about how worthwhile it was for your child?—Group 2 only*]
**Communication about CMV Testing**
20. Thinking back to the initial diagnosis of congenital CMV in your child, what kind of information or help would have been most useful to you then? Who would be the best person or source to give new parents that information?
21. And then later as your child entered the follow up visits and testing, what kind of information or help would have been most useful to you then? Who would be the best person or source to give new parents that information?
22. Based on your experience, do you think newborn CMV testing should be offered to all new parents? If you think so, what concerns would you have about that? Also, should the testing be mandatory or should it require parental permission? Why or why not?
23. What are the three most important things you would want to say to other parents about having a positive CMV test result?
a. Does what you need to tell parents differ as the child gets older? How so?
b. Where do you tell them to get more information if they need it?
c. Are there good Internet resources you would send them to? Which ones?
24. Who do you think does a better job helping with the information needs of parents of newly diagnosed children, other parents, doctors, nurses, others? What is that they do better?
**Closing Thoughts**
25. Before we finish up today, I’d like to ask if there are any other topics or issues, positive or negative, that you would like to mention?
Thank you so much. Your thoughts and insights today have been very helpful to this project. We will be interviewing some other parents as well. After that we will be conducting a mail survey to collect more detailed information from you. If you do not want to be contacted about that survey, please let one of the CMV staff people know that. One of them will also make sure you have parking tokens and are signed up to receive the $25 incentive as a token of appreciation for your interest. Thank you again and drive safely going home.

## Appendix B. Parent Focus Group Guide

**Group 3—Parents of children born with CMV who were asymptomatic and did not develop sequelae**
**Consent**
Good afternoon, my name is _____________. I want to thank you in advance for taking time from your busy schedule to participate in this focus group. This research is being sponsored by the Centers for Disease Control and Prevention. The information you provide today will be extremely useful to other parents and to professionals working on congenital CMV. When one of the CMV staff members called and asked you to participate today you were told that this focus group is entirely voluntary, that no LAST names will be used today or in our reporting of the data, and that if there are any questions you do not want to answer or feel are too sensitive you are free to not reply or to write down your comments and hand them to me after the session if you’d prefer. In fact, you are free to leave the focus group session at any time. We will be recording today’s session, so please try to speak one at a time. If each of you could signify by saying your FIRST NAME ONLY and YES you understand how your privacy is being protected we can proceed. [The moderator will go around the table and ask each participant to state their agreement with the informed consent]. I’ll stop for a minute to see if there are any questions- anyone?
**Purpose**
As I said my name is _________ and I am working with Dr. Gail Demmler-Harrison and her CMV team on a project to help us better understand the kinds of positive and negative mental, emotional, financial, and family issues that parents experience when their child is first tested for congenital CMV and in the following critical years of development as follow-up tests may be completed. One of the best ways for us to do that is to talk directly to people like you who know a lot about those experiences. I will be asking you to share your thoughts, feelings, and opinions about these experiences. Please remember that there are no right or wrong answers—we just want to know about your own thoughts, whatever they may be. I want to encourage you all to participate in an open discussion. From time to time I may move the discussion so we can cover all of our topics tonight. Let me stop and ask if there are any questions so far.
The last important thing to know is that each of you in this group share the experience of having a child test positive for congenital CMV. [Moderator to note this group is Parents whose child was asymptomatic, C-CMV positive, but did not develop sequelae]
**Procedures**
- Session should last about an hour to an hour and a half - This is a group discussion, so you don’t have to wait to be called on - Secure—when we report about these conversations later, not even your FIRST name will be used in the report. - The restrooms are ____. Feel free to get up at any time during our discussion. - Please help yourself to the refreshments - Any other questions before we start? Okay, let’s get started.
**Warm Up and Opening Question (5 min)**
1. First, I’d like to go around the table and have each of you tell me your FIRST name only and one of your memories from the first 5 years of life of your child who was born infected with CMV. [Moderator will encourage any kind of memory sharing they prefer].
**Negative Psychosocial Impacts of CMV Test and Follow-up Testing**
(Note: moderator will rotate the positive and negative impacts sections among the focus group sessions to minimize any order bias].
2. As you all know raising a child can have its ups and downs. But we are particularly interested in your memory of experiences you had when your child was first diagnosed positive for congenital CMV. So take a minute and think back now to that time, when your child was an infant and you received this testing news. Tell me about your first reactions?
3. After the positive CMV test results were given to you, what would you say were the first few things you wanted to know? Was anyone able to answer those questions for you?
4. In those first few days tell me how you felt about yourself in regards to the positive test? [Probe for feeling alone, feeling responsible, being scared, feeling overwhelmed, no major concerns, not a problem].
5. In the first few days that followed the positive CMV test results, tell me about any kinds of family or marital stress you experienced. [Moderator to probe for spousal issues like blaming, concerns about how my other children and how they would cope, worries about what my family would think/react].
6. Can you tell me what you thought about in terms of financial issues related to the positive test results? Did you worry about anything, what kinds of things? [Probe for concerns about enough money to have necessary follow-up tests done, concerns about health insurance coverage, worries about how I can care for this child and my other children].
7. Now let’s talk a little about the years following the initial test results. All of you participated in the follow-up clinic for some period of time. After the first few months, what kinds of new concerns or stressful issues surfaced? [Probe for stressors specific to self, marriage/spouse, and parenting skills/efficacy].
8. After the first few months, what kinds of new concerns or stressful issues surfaced related to your family, friends, and other social networks (e.g., church)?
9. Now thinking about the medical professionals you met with over those first few years, were there any additional stressors from those meetings? What kinds of things frustrated you? What could have been explained better? Thinking about new parents today, how would you explain some of the things that frustrated you?
10. Now thinking about those extra doctor visits and follow-up tests that your child went through, what kinds of stress or negative impacts did those activities or tests have on you and your family? [Probe for transportation hassles of getting to appointments, concerns about time off of work to get to appointments, costs of travel, difficulty with child care for other children].
11. Now let’s talk about your interactions with your child. Thinking back over those first few years, how would you characterize your relationship with your child? [Probe for stressors related to child behaviors, lack of affection, too much affection/attention, over-protectiveness].
12. If you have other children, did you feel that your relationship with your CMV+ child was different in some ways due to the CMV? How so?
13. As your child got older, what kinds of concerns did they have about the doctor visits and follow-up tests? What could have been done or said to you to improve that experience for you and for your child?
**Positive Psychosocial Impacts**
(Note: moderator will rotate the positive and negative impacts sections among the focus group sessions to minimize any order bias].
Now let’s switch gears and talk about some of the positive aspects of having the CMV test done and the knowledge that came with that.
14. Thinking back to initial diagnosis of congenital CMV infection in your newborn child, what would you say was the most positive aspect of getting those results?
15. Now thinking about the following few years and the doctor visits and follow up tests that you and your child went through, what would you say are the best things about having done those visits and tests? [Probe for the kinds of concerns that the follow-up testing minimized].
a. What would you say were the worst things about having done those visits and tests?
16. All in all, tell me about your thinking about the value of CMV testing and follow-up testing. Were the tests worth the troubles, stress, and issues that the testing caused for you as a parent?
a. What about how worthwhile it was for your child?
**Communication about CMV Testing**
17. What are the three most important things you would want to say to other parents about having a child born with CMV infection?
a. Does what you need to tell parents differ as the child gets older? How so?
b. Where do you tell them to get more information if they need it?
c. Are there good Internet resources you would send them to? Which ones?
18. Who do you think does a better job helping with the information needs of parents of newly diagnosed children, other parents, doctors, nurses, others? What is that they do better?
19. Based on your experience, do you think newborn CMV testing should be offered to all new parents? If you think so, what concerns would you have about that? Also, should the testing be mandatory or should it require parental permission? Why or why not?
20. What are the three most important things you would want to say to other parents about having a positive CMV test result?
a. Does what you need to tell parents differ as the child gets older? How so?
b. Where do you tell them to get more information if they need it?
c. Are there good Internet resources you would send them to? Which ones?
**Closing Thoughts**
21. Before we finish up today, I’d like to ask if there are any other topics or issues, positive or negative, that you would like to mention?
Thank you all so much. Your thoughts and insights today have been very helpful to this project. We will be conducting group meetings with other parents and interviewing some parents as well. After that we will be conducting a mail survey to collect more detailed information from all of you. If you do not want to be contacted about that survey, please let one of the CMV staff people know that. One of them will also make sure you have parking tokens and are signed up to receive the $25 incentive as a token of appreciation for your interest. Thank you again and drive safely going home.
